# NLRP3 Inflammasome Inhibitor BAY-117082 Reduces Oral Squamous Cell Carcinoma Progression

**DOI:** 10.3390/ijms222011108

**Published:** 2021-10-15

**Authors:** Sarah Adriana Scuderi, Giovanna Casili, Rossella Basilotta, Marika Lanza, Alessia Filippone, Gabriele Raciti, Ivana Puliafito, Lorenzo Colarossi, Emanuela Esposito, Irene Paterniti

**Affiliations:** 1 Department of Chemical, Biological, Pharmaceutical and Environmental Sciences, University of Messina, 6 Viale Ferdinando Stagno d’Alcontres 31, 98166 Messina, Italy; sarahadriana.scuderi@unime.it (S.A.S.); gcasili@unime.it (G.C.); rossella.basilotta@unime.it (R.B.); mlanza@unime.it (M.L.); afilippone@unime.it (A.F.); ipaterniti@unime.it (I.P.); 2 IOM Ricerca Srl, Via Penninazzo 11, 95029 Catania, Italy; gabriele.raciti@grupposamed.com; 3 Istituto Oncologico del Mediterraneo, Via Penninazzo 7, 95029 Catania, Italy; ivana.puliafito@grupposamed.com (I.P.); lorenzo.colarossi@grupposamed.com (L.C.)

**Keywords:** oral cancer, oral squamous cell carcinoma (OSCC), NOD-like receptor family pyrin domain containing 3 inflammasome (NLRP3), apoptosis

## Abstract

Oral cancer is one of the most common human malignancies, and its incidence is increasing worldwide. In particular, oral squamous cell carcinoma (OSCC) is characterized by high rates of proliferation, invasiveness, and metastasis. Currently, standard treatment for OSCC includes surgical removal, chemotherapy, and radiotherapy; however, the survival rate of patients with OSCC remains low, thus new therapies are needed. It has been proven that excessive NLRP3 inflammasome activation and apoptosis alteration may contribute to oral cancer progression. This study aimed to investigate the effect of BAY-117082, an NLRP3 inflammasome inhibitor, in an in vitro and in vivo xenograft model of oral cancer. In vitro results revealed that BAY-117082 at concentrations of 5, 10, and 30 µM was able to reduce OSCC cell viability. BAY-117082 at higher concentrations significantly reduced NLRP3, ASC, caspase-1, IL-1β, and IL-18 expression. Moreover, Bax, Bad, and p53 expression were increased, whereas Bcl-2 expression was reduced. Furthermore, the in vivo study demonstrated that BAY-117082 at doses of 2.5 and 5 mg/kg significantly decreased subcutaneous tumor mass, and also reduced NLRP3 inflammasome pathway activation. Therefore, based on these results, the use of BAY-117082 could be considered a promising strategy to counteract oral cancer progression, thanks its ability to modulate the NLRP3 inflammasome and apoptosis pathways.

## 1. Introduction

Oral cancer is one of the most common malignancies in the world [1]. Oral cancer commonly occurs in older individuals, although a troubling number of these malignancies have also been documented in younger adults [1]. Oral squamous cell carcinoma (OSCC) represents more than 90% of all oral cancers [2]. Smoking and excessive alcohol consumption are the major risk factors for the development of OSCC [2]. The clinical manifestations of OSCC can negatively influence the quality of life of patients, causing dysfunction in talking and swallowing, sensory impairment, and chronic pain [3]. Currently, the treatment for oral cancer includes surgical resection of the tumor followed by postoperative adjuvant therapy [3]. However, the survival rate for patients with OSCC still remains low [1]; consequently, identifying new therapeutic targets and new molecules able to reduce or arrest the progression of oral cancer is an important goal in cancer research. Evidence demonstrates that excessive innate immune system activation may contribute to various human malignancies, including oral cancer [4,5]. The nucleotide-binding domain leucine-rich repeat-containing receptors (NLRs) are a family of intracellular innate immune receptors that play a key role in the inflammatory response and the innate immune system [6]. In response to various intracellular stimuli, NLRs are activated, inducing the assembly of multiprotein complexes known as inflammasomes [7]. As a member of the inflammasome family, NOD-like receptor family pyrin domain containing 3 (NLRP3) is the most studied and best characterized inflammasome, and has been shown to be involved in several pathologies, including neurodegenerative diseases, atherosclerosis, and cancer [7,8]. The NLRP3 inflammasome, through apoptosis-associated speck-like protein (ASC), an adapter protein, activates caspase-1, which in turn promotes the release of pro-inflammatory cytokines interleukin (IL)-1β and IL-18, contributing to the innate immune response [8]. However, excessive and aberrant NLRP3 inflammasome activation may lead to cancer progression [8]. 

Recent studies demonstrated that the NLRP3 inflammasome pathway is upregulated in OSCC animal models and OSCC patients [4], suggesting its involvement in oral cancer pathogenesis. in the oral carcinogenesis mechanism, apoptosis also plays a key role [9]. Apoptosis is a programmed cell death process that promotes the removal of stressed, damaged, transformed, or infected cells [9]. However, abnormalities in the apoptosis pathway can result in various human diseases, including cancer [10]. Thus, more in-depth studies of the mechanism of apoptosis could provide a new opportunity to discover and develop novel agents that could increase the sensitivity of cancer cells to apoptosis or reset their apoptotic threshold [11]. Recent studies demonstrated that BAY 11-7082, a sulfonic derivative, is a strong inhibitor of NLRP3 inflammasome thanks its ability to suppress the ATPase activity of NLRP3, required for its activation [6,12,13]. BAY 11-7082 exerts several pharmacological activities, including anticancer, neuroprotective, and anti-inflammatory effects [12,14,15]. Furthermore, it has been proven that BAY-117082 is able to modulate the apoptosis process [16,17]. Therefore, considering the keys role of NLRP3 inflammasome and the apoptosis pathway in oral cancer pathogenesis, the aim of this study was to investigate the potential effect of BAY 11-7082, an NLRP3 inflammasome inhibitor, on reducing oral cancer growth in an in vitro and in vivo xenograft model of OSCC. 

## 2. Results 

### 2.1. In Vitro Results

#### 2.1.1. BAY-117082 Reduced OSCC Cell Viability

**Figure 1:**
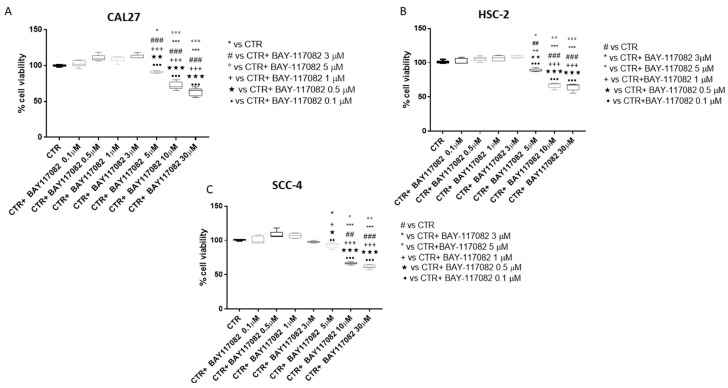
Effect of BAY-117082 on CAL27, HSC-2 and SCC-4 cell viability. MTT results demonstrate that BAY-117082 at higher concentrations significantly reduced CAL27, HSC-2, and SCC-4 cell viability. (**A**) * *p* < 0.05 vs. CTR; *** *p* < 0.001 vs. CTR; ### *p* < 0.001 vs. CTR + BAY-117082 3 µM; °°° *p* < 0.001 vs. CTR + BAY-117082 5 µM; +++ *p* < 0.001 vs. CTR + BAY-117082 1 µM; ••• *p*< 0.001 vs. CTR + BAY-117082 0.1 µM; ★★ *p* < 0.01, ★★★ *p* < 0.001 vs. CTR + BAY-117082 0.5 µM; (**B**) ## *p* < 0.01, ### *p* < 0.001 vs. CTR; * *p* < 0.05, *** *p* < 0.001 vs. CTR + BAY-117082 3 µM; °° *p* < 0.01, °°° *p* < 0.001 vs. CTR + BAY-117082 5 µM; ++ *p* < 0.01, +++ *p* < 0.001 vs. CTR + BAY-117082 1 µM; ★★ *p* < 0.01, ★★★ *p* < 0.001 vs. CTR + BAY-117082 0.5 µM; ••• *p* < 0.001 vs. CTR + BAY-117082 0.1 µM; (**C**) ## *p* < 0.01, ### *p* < 0.001 vs. CTR; * *p* < 0.05, *** *p* < 0.001 vs. CTR + BAY-117082 3 µM; ° *p* < 0.05, °° *p* < 0.01 vs. CTR + BAY-117082 5 µM; + *p* < 0.05 +++ *p* < 0.001 vs. CTR + BAY-117082 1 µM; ★ *p* < 0.05, ★★★ *p* < 0.001 vs. CTR+BAY-117082 0.5 µM; •• *p* < 0.01, ••• *p* < 0.001 vs. CTR + BAY-117082 0.1 µM.

MTT assay was used to assess CAL27, HSC-2, and SCC-4 cell viability following 24 h of treatment with BAY-117082 at different concentrations (0.1, 0.5, 1, 3, 5, 10, and 30 µM). Our results show that BAY-117082 treatment only at concentrations of 5, 10, and 30 µM significantly reduced CAL27, HSC-2, and SCC-4 cell viability in the same way, as shown in Figure 1. 

Based on the MTT results, we decided to investigate in another analysis only BAY-117082 at concentrations of 5, 10, and 30 µM, because these represented the most cytotoxic concentrations. 

Furthermore, since BAY-117082 showed similar effects on cell viability in all three cell lines, we decided to continue to investigate its effect on only the CAL27 cell line, because it represented a frequently used cell line in the field of OSCC [18,19].

#### 2.1.2. BAY-117082 Reduced NLRP3 Inflammasome Pathway Activation

**Figure 2:**
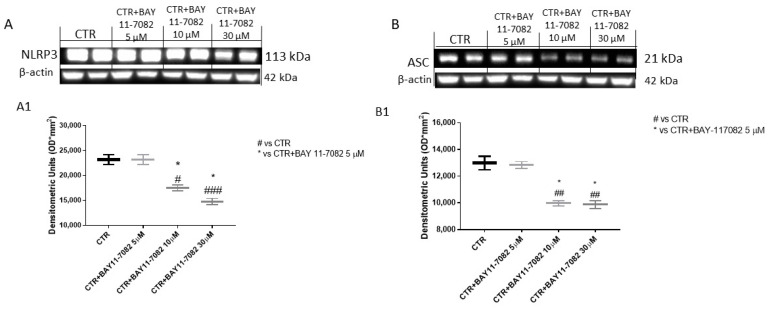
Effect of BAY-117082 on NLRP3 inflammasome and ASC expression. Blots reveal that treatment with BAY-117082 at concentrations of 10 and 30 µM was able to reduce NLRP3 and ASC expression compared to control group. Data are representative of at least three independent experiments. (**A**) # *p* < 0.05 vs. CTR; ### *p* < 0.001 vs. CTR; * *p* < 0.05 vs. CTR + BAY-117082 10 µM. (**B**) ## *p* < 0.01 vs. CTR; * *p* < 0.05 vs. CTR + BAY-117082 10 µM.

**Figure 3:**
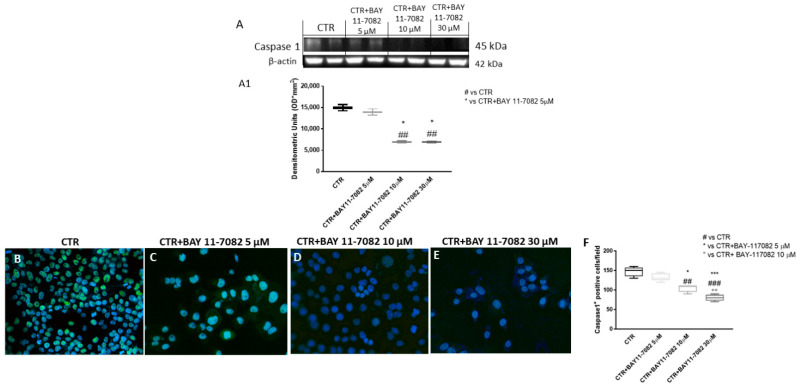
(**A**–**E**) Effect of BAY-117082 on caspase-1 expression. Blots reveal that BAY-117082 treatment at concentrations of 10 and 30 µM was able to reduce caspase-1 expression compared to control group. (**F**) Data were also confirmed by immunofluorescence assay. Data are representative of at least three independent experiments. (**A**) ## *p* < 0.01 vs. CTR; * *p* < 0.05 vs. CTR + BAY-17082 5 µM. (**F**) ## *p* < 0.01 vs. CTR; ### *p* < 0.001 vs. CTR; * *p* < 0.05 vs. CTR + BAY-17082 5 µM; *** *p* < 0.001 vs. CTR + BAY-17082 5 µM; °° *p* < 0.01 vs. CTR + BAY-117082 10 µM.

Inflammasome is a cytoplasmic multi-protein complex that regulates innate immunity response through ASC and caspase-1 activation [6]. NLRP3 inflammasome is reported to be abnormally expressed and activated in malignancies such as cancer, favoring its progression [20]. Therefore, in this study we decided to investigate the NLRP3 inflammasome pathway by Western blot analysis in CAL27 cell lysates. Our results show that the control group was characterized by elevated NLRP3, ASC, and caspase-1 expression, while BAY-117082 treatment only at concentrations of 10 and 30 µM significantly reduced their expression compared to control (Figure 2A,B and Figure 3A). The data for caspase-1 were also confirmed by immunofluorescence staining, showing that treatment with BAY-117082 only at concentrations of 10 and 30 µM significantly reduced its expression compared to control (Figure 3B–E; also see caspase-1 positive cell score, Figure 3F).

**Figure 4:**
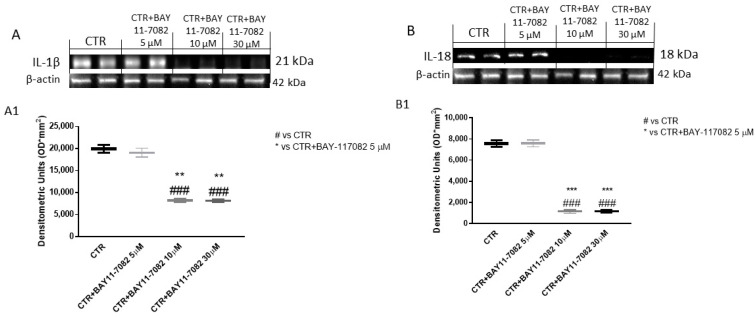
Effect of BAY-117082 on IL-1β and IL-18 expression. Blots reveal that BAY-117082 treatment at concentrations of 10 and 30 µM was able to reduce IL-1β and IL-18 expression compared to control group. Data are representative of at least three independent experiments. (**A**) ### *p* < 0.001 vs. CTR; ** *p* < 0.01 vs. CTR + BAY-117082 5 µM. (**B**) ### *p* < 0.001 vs. CTR; *** *p* < 0.001 vs. CTR + BAY-117082 5 µM.

Once activated, NLRP3 inflammasome induces the release of pro-inflammatory cytokines IL-1β and IL-18, which can contribute to cancer progression [21,22]. Thus, in this study we evaluated IL-1β and IL-18 expression by Western blot analysis in CAL27 cell lysates. Our results show that treatment with BAY-117082 only at concentrations of 10 and 30 µM significantly reduced their expression compared to the control group (Figure 4). 

#### 2.1.3. BAY-117082 Modulated Apoptosis Pathway

**Figure 5:**
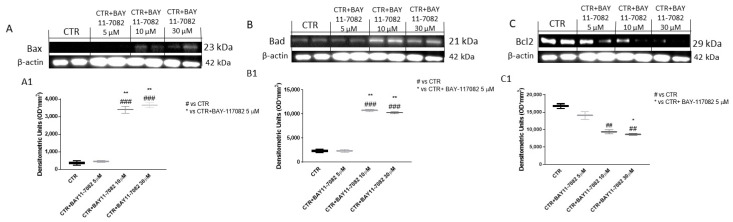
Effect of BAY-117082 on apoptosis pathway. Blots reveal that with BAY-117082 treatment at concentrations of 10 and 30 µM (**A**,**B**) Bax and Bad expression was increased and (**C**) Bcl2 expression was significantly reduced compared to control group. Data are representative of at least three independent experiments. (**A**) ### *p* < 0.001 vs. CTR; ** *p* < 0.01 vs. CTR + BAY-117082 5 µM. (**B**) ### *p* < 0.001 vs. CTR; ** *p* < 0.01 vs. CTR + BAY-117082 5 µM. (**C**) ## *p* < 0.01 vs. CTR; * *p* < 0.05 vs. CTR + BAY-117082 5 µM.

Apoptosis is a physiological process of programmed cell death that is essential for normal tissue development and cell hemostasis [9]. Dysregulation in the apoptotic process has been associated with several diseases, including oral cancer [9]. Therefore, we decided to investigate the apoptotic markers Bax, Bad, and Bcl2 by Western blot analysis in CAL27 cell lysates. Our results show that treatment with BAY-117082 only at concentrations of 10 and 30 µM significantly increased pro-apoptotic Bax and Bad expression compared to the control group (Figure 5A,B), while anti-apoptotic Bcl2 expression was significantly reduced following BAY-117082 treatment compared to control (Figure 5C). 

Recent studies highlighted the role of pro-apoptotic p53 protein in modulating various cellular processes such as metabolism, metastasis, and communication within the tumor microenvironment [23]. p53 deficiency and altered apoptosis can enhance the initiation or progression of cancer [23]. Thus, in this study we decided to investigate p53 expression by immunofluorescence assay in CAL27 cells.

**Figure 6:**
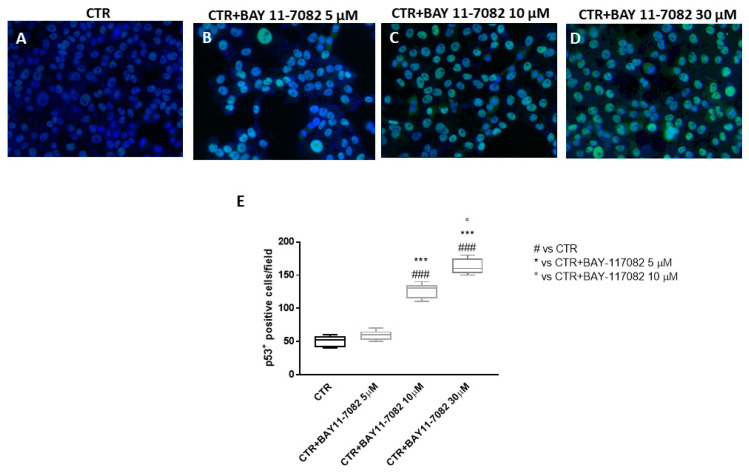
Effect of BAY-117082 on p53 expression. (**A**–**D**) Immunofluorescence assay revealed that BAY-117082 treatment at concentrations of 10 and 30 µM was able to significantly increase p53 expression compared to control group in CAL27 cells. Data are representative of at least three independent experiments. (**E**) ### *p* < 0.001 vs. CTR; *** *p* < 0.001 vs. CTR + BAY-117082 5 µM; ° *p* < 0.05 vs. CTR + BAY-117082 10 µM.

Our results demonstrate that the control group was characterized by low p53 expression, while treatment with BAY-117082 at concentrations of 10 and 30 µM significantly increased pro-apoptotic p53 expression (Figure 6A–D; also see p53 positive cell score, Figure 6E). 

### 2.2. In Vivo Results

#### 2.2.1. BAY-117082 Reduced Tumor Growth

**Figure 7:**
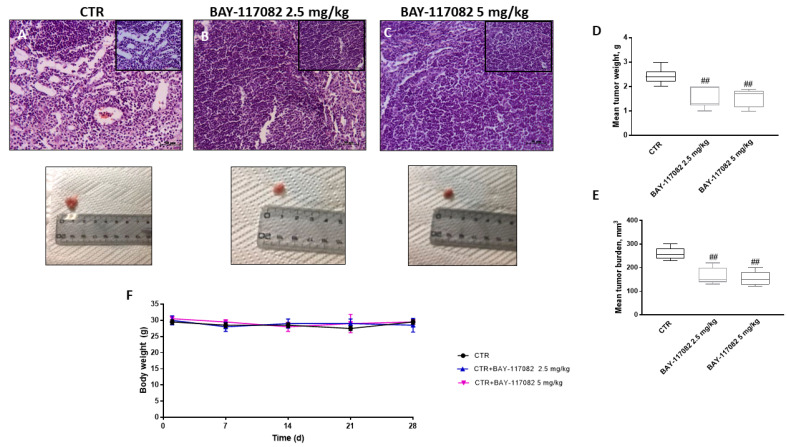
Effect of BAY-117082 on tumor growth in CAL27 xenograft model. (**A**–**C**) BAY-117082 treatment at doses of 2.5 and 5 mg/kg was able to significantly reduce subcutaneous tumor mass compared to control group. (**D**,**E**) Additionally, BAY-117082 reduced tumor burden and tumor weight, (**F**) without important weight differences between animals. Data are representative of at least three independent experiments. Sections were observed and photographed at 20× and 40× magnification. (**D**) ## *p* < 0.01 vs. CTR; (**E**) ## *p* < 0.01 vs. CTR.

To evaluate the effect of BAY-117082 on the growth of OSCC cells in vivo, the CAL27 xenograft model was established in nude mice. In this context, our results show that treatment with BAY-117082 at doses of 2.5 and 5 mg/kg significantly reduced subcutaneous tumor mass and neutrophilic infiltration in a dose-dependent manner (Figure 7A–C). Moreover, BAY-117082 at these two doses significantly reduced tumor burden and tumor weight in a dose-dependent manner compared to the control group (Figure 7D,E). During the course of treatment, no important change in the animals’ weight was seen (Figure 7F).

#### 2.2.2. BAY-117082 Reduced NLRP3 Inflammasome Activation in CAL27 Xenograft Model

**Figure 8:**
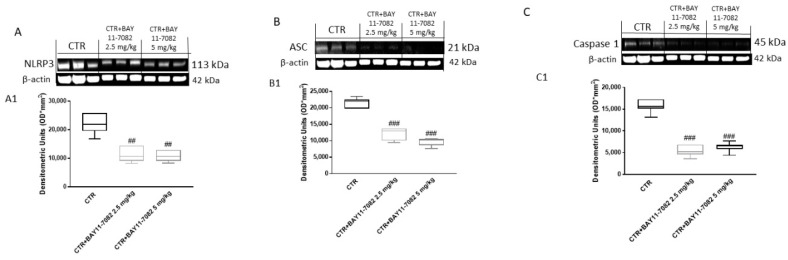
Effect of BAY-117082 on NLRP3 inflammasome pathway in CAL27 xenograft model. Blots reveal that treatment with BAY-117082 at doses of 2.5 and 5 mg/kg was able to reduce NLRP3, ASC, and caspase 1 expression compared to control group. Data are representative of at least three independent experiments. (**A**) ## *p* < 0.01 vs. CTR; (**B**) ### *p* < 0.001 vs. CTR; (**C**) ### *p* < 0.001 vs. CTR.

To confirm the effect of BAY-117082 on the NLRP3 inflammasome pathway, we decided to also investigate this pathway in the xenograft model by Western blot analysis. The results show that the control group was characterized by high expression of NLRP3, ASC, and caspase-1; however, treatment with BAY-117082 at doses of 2.5 and 5 mg/kg was able to significantly reduce their expression (Figure 8). 

**Figure 9:**
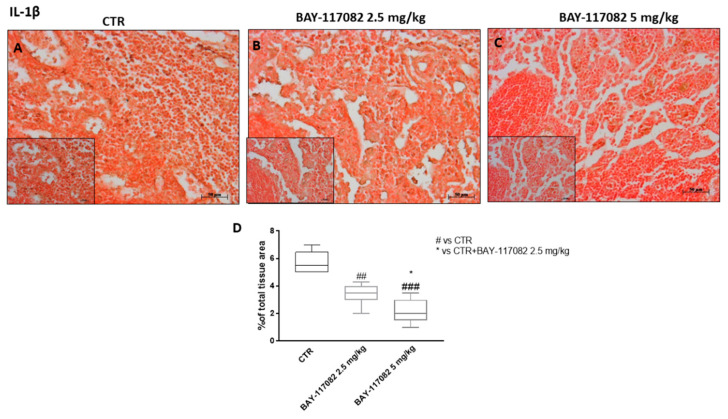
Effect of BAY-117082 on IL-1β expression in CAL27 xenograft model. Immunohistochemistry assay revealed that (**A**) control group was characterized by high IL-1β expression, and (**B**,**C**) treatment with BAY-117082 at doses of 2.5 and 5 mg/kg significantly reduced its expression. Data are representative of at least three independent experiments. Sections were observed and photographed at 20× and 40× magnification. (**D**) ## *p* < 0.01 vs. CTR; ### *p* < 0.001 vs. CTR; * *p* < 0.05 vs. CTR + BAY-117082 2.5 mg/kg.

Moreover, we decided to investigate, by immunohistochemistry staining, the expression of IL-1β, a pro-inflammatory cytokine related to cancer progression. Our results show that the control group was characterized by marked expression of IL-1β, while treatment with BAY-117082 at doses of 2.5 and 5 mg/kg was able to reduce its expression in a dose-dependent manner (Figure 9), confirming the previously obtained in vitro data. 

#### 2.2.3. BAY-117082 Modulated NF-κB/IκB-α Pathway in CAL27 Xenograft Model

**Figure 10:**
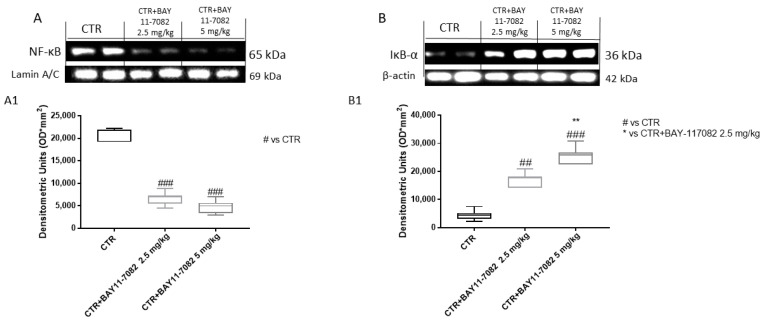
Effect of BAY-117082 on NF-κB/IκB-α pathway in CAL27 xenograft model. Blots reveal that (**A**) BAY-117082 treatment reduced NF-κB expression compared to control group, and (**B**) IκB-α expression was restored in a dose-dependent manner. Data are representative of at least three independent experiments. (**A**) ### *p* < 0.001 vs. CTR. (**B**) ## *p* < 0.01 vs. CTR; ### *p* <0.001 vs. CTR; ** *p* < 0.01 vs. CTR + BAY-117082 2.5 mg/kg.

Accumulating evidence has suggested that the nuclear factor-κB (NF-κB) signaling pathway plays a critical role in oral carcinogenesis [24,25]. It has been demonstrated that NF-κB pathway activation promotes initiation and progression of oral cancer, increasing metastasis and cell invasion [25]. Therefore, we decided to evaluate the NF-κB/IκB-α pathway by Western blot analysis. Our data reveal that BAY-117082 at doses of 2.5 and 5 mg/kg was able to reduce NF-κB translocation into the nucleus and restore IκB-α cytosolic expression in a dose-dependent manner compared to the control group (Figure 10).

#### 2.2.4. BAY-117082 Modulated CD4, CD8, and CD30 Expression in CAL27 Xenograft Model

**Figure 11:**
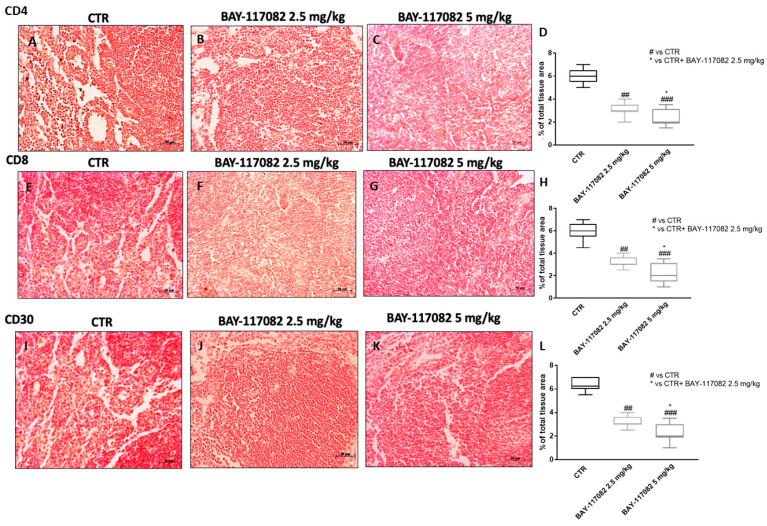
Effect of BAY-117082 on (**A**–**D**) CD4, (**E**–**H**) CD8, and (**I**–**L**) CD30 expression in CAL27 xenograft model. Immunohistochemical staining revealed that control group was characterized by high CD4, CD8, and CD30 expression, whereas treatment with BAY-117082 significantly reduced their expression in a dose-dependent manner. Data are representative of at least three independent experiments. Sections were observed and photographed at 20× magnification. (**D**) ## *p* < 0.01 vs. CTR; ### *p* < 0.001 vs. CTR; * *p* < 0.05 vs. CTR + BAY-117082 2.5 mg/kg. (**H**) ## *p* < 0.01 vs. CTR; ### *p* < 0.001 vs. CTR; * *p* < 0.05 vs. CTR + BAY-117082 2.5 mg/kg. (**L**) ## *p* < 0.01 vs. CTR; ### *p* < 0.001 vs. CTR; * *p* < 0.05 vs. CTR + BAY-117082 2.5 mg/kg.

Cancer development and its response to therapy are strongly influenced by innate and adaptive immunity, which can promote or attenuate tumorigenesis with opposite effects on the therapeutic outcome [26]. It has been demonstrated that aberrant immune system activation can influence the carcinogenesis process [26]. Therefore, in this study we decided to evaluate the effect of BAY-117082 on the immune system by evaluating CD4, CD8, and CD30 expression. Our data demonstrate that BAY-11082 treatment at doses of 2.5 and 5 mg/kg significantly reduced CD4, CD8, and C30 levels compared to the control group in a dose-dependent manner (Figure 11).

#### 2.2.5. BAY-117082 Modulated Apoptosis in CAL27 Xenograft Model

**Figure 12:**
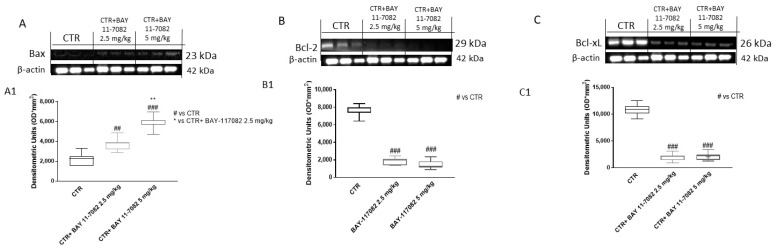
Effect of BAY-117082 on apoptosis pathway in CAL27 xenograft model. Blots reveal that (**A**) BAY-117082 treatment at doses of 2.5 and 5 mg/kg was able to increase Bax expression, and (**B**,**C**) Bcl2 and Bcl-xL expression was significantly reduced compared to control group. Data are representative of at least three independent experiments. (**A**) ## *p* < 0.01 vs. CTR; ### *p* < 0.001 vs. CTR; ** *p* < 0.01 vs. CTR + BAY-117082 2.5 mg/kg. (**B**) ### *p* < 0.001 vs. CTR; (**C**) ### *p* < 0.001 vs. CTR.

Apoptosis plays a key role in cancer progression, therefore targeting apoptosis could be a relevant therapeutic approach in anti-cancer drug development [10]. Thus, we decided to also investigate in the xenograft model the effect of BAY-117082 on the apoptosis pathway, evaluating the apoptotic markers Bax, Bcl2, and Bcl-xL by Western blot analysis. Our results demonstrate that treatment with BAY-117082 at doses of 2.5 and 5 mg/kg was able to increase pro-apoptotic Bax protein expression and reduce anti-apoptotic Bcl2 and Bcl-xL protein expression in a dose-dependent manner compared to the control group (Figure 12), confirming the previously obtained in vitro data.

#### 2.2.6. BAY-117082 Reduced Ki-67 Expression in CAL27 Xenograft Model

**Figure 13:**
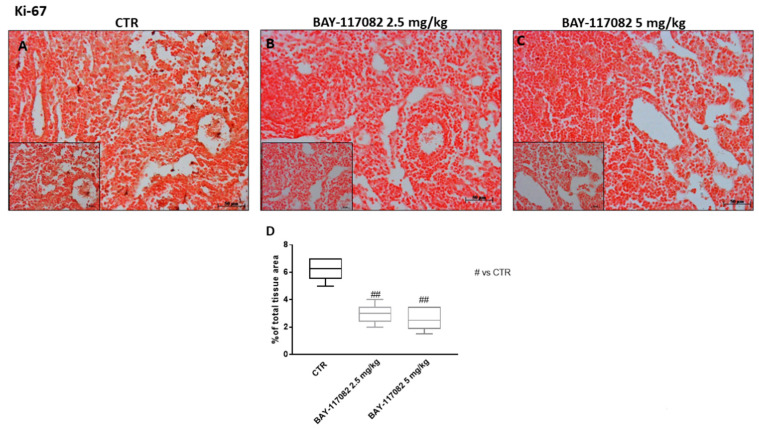
Effect of BAY-117082 on Ki-67 expression in CAL27-xenograft model. Immunohistochemistry assay revealed that (**A**) control group was characterized by marked Ki-67 expression, and (**B**,**C**) treatment with BAY-117082 at doses of 2.5 and 5 mg/kg significantly reduced its expression. Data are representative of at least three independent experiments. Sections were observed and photographed at 20× and 40× magnification. (**D**) ## *p* < 0.01 vs. CTR.

Ki-67 is a DNA-binding nuclear protein involved in cell proliferation [27], and is widely used as a prognostic and predictive indicator of cancer progression [28]. Clinically, Ki-67 has also been shown to correlate with metastasis and the clinical stage of tumors [27]. Therefore, in this study we decided to investigate Ki-67 expression as a marker of cancer cell proliferation by immunohistochemistry staining. Our data show that treatment with BAY-117082 at doses of 2.5 and 5 mg/kg was able to significantly reduce Ki-67 expression compared to the control group (Figure 13A–C).

## 3. Discussion 

Oral squamous cell carcinoma (OSCC), the most common oral cancer, arises from the mucosal lining of the oral cavity, with an incidence of 450,000 new cases per year [2]. Major risk factors for OSCC development include smoking and excessive alcohol consumption, but other factors such as human papillomavirus (HPV), nutritional deficiencies, and genomic alterations have also been associated [2,29]. Clinical treatment of oral cancer includes surgical resection, chemotherapy, and radiotherapy, however, additional therapies are needed [1]. Previous studies have demonstrated that aberrant innate immune system activation may promote oral carcinogenesis [21,30]. Inflammasomes are multi-protein complexes that are involved in the innate immune system and the inflammatory response [31]. Inflammasomes belong to a larger family of receptors known as pattern recognition receptors (PRRs); their function is to recognize pathogen- or danger-associated molecular patterns (PAMPs or DAMPs), causing the activation, maturation, and production of pro-inflammatory cytokines [32]. NLRP3 inflammasome is one of the most studied inflammasomes belonging to the NLR protein family [7]. 

Although the pathophysiology of oral cancer remains unclear, in vivo and in vitro studies have demonstrated that aberrant and excessive NLRP3 inflammasome activation significantly contributes to the initiation and progression of oral cancer [5,20]. Moreover, it has been proven that cell apoptosis also has a predominant role in oral cancer [10]. Specifically, evidence suggests that dysregulation in the apoptosis process may induce cancer progression, promoting cancer cell survival [9,33]. Recently, more attention has been given to the potential application of NLRP3 inhibitors for the treatment of cancer. BAY-117082, a sulfonic derivate, is a strong inhibitor of NLRP3 inflammasome, with neuroprotective and anti-inflammatory effects [6,14,16]. An important study conducted by Chen et al. demonstrated that BAY-117082 exerts an anti-tumor effect through mitochondrial pathway modulation, suggesting its possible use as a promising treatment for cancer [17]. Therefore, based on the key roles of the NLRP3 inflammasome and apoptosis pathways in oral carcinogenesis, in this study we decided to evaluate the beneficial effect of BAY-117082 in in vitro and in vivo xenograft models of oral cancer. 

First of all, we evaluated the cytotoxic effect of BAY-117082 at different concentrations in an in vitro model of OSCC using CAL27, HSC-2, and SCC-4 cell cultures. Clearly, our results demonstrate that BAY-117082 treatment only at higher concentrations significantly reduced CAL27, HSC-2, and SCC-4 cell viability in the same way. 

Many papers have proven that NLRP3 inflammasome plays a key role in oral carcinogenesis [5,21]. NLRP3 inflammasome regulates innate immunity response through ASC and caspase-1 activation, consequently promoting the inflammatory response [6]. 

However, recent studies have demonstrated that excessive and aberrant NLRP3 inflammasome activation may contribute to oral cancer progression [4,20]. Thus, we decided to investigate the effect of BAY-117082 on the NLRP3 inflammasome pathway, showing that BAY-117082 at higher concentrations was able to significantly reduce NLRP3, ASC, and caspase-1 expression compared to the control group in CAL27 cell lysates. 

Once activated in response to intracellular stimuli, NLRP3 inflammasome promotes the proteolytic processing of pro-IL-1β and pro-IL-18 into their bioactive forms IL-1β and IL-18, respectively [34]. Overexpression of pro-inflammatory cytokines such as IL-1β and IL-18 may promote cancer progression, increasing an excessive inflammatory response [22]. Our results demonstrate that the control group was characterized by marked expression of IL-1β and IL-18, whereas treatment with BAY-117082 at higher concentrations significantly reduced their expression compared to the control. 

Among the mechanisms involved in cancer pathogenesis, apoptosis also has a fundamental role [33,35]. Programmed cell death occurs through two main signaling pathways, one defined as intrinsic and the other extrinsic [35]. The intrinsic pathway is regulated by the balance of two proteins, Bax and Bcl-2; Bax has a pro-apoptotic role, while Bcl-2 exerts anti-apoptotic activity. On the other hand, the extrinsic pathway is regulated by caspase cascade activation, which stimulates chromatin fragmentation [36,37]. Apoptosis appears to be a very interesting and complex phenomenon, thus further investigation into its mechanism is needed to discover and develop novel agents that could increase the sensitivity of cancer cells to programmed cell death [38]. Therefore, in this study we decided to investigate the effect of BAY-117082 on the apoptosis pathway, evaluating the pro-apoptotic markers Bax, Bad, and p53 and anti-apoptotic marker Bcl2. Our results show that the control group was characterized by low expression of Bax, Bad, and p53, whereas treatment with BAY-117082 at higher concentrations significantly increased their expression. Moreover, BAY-117082 treatment significantly reduced anti-apoptotic marker Bcl2 expression compared to the control group. 

Despite the promising results obtained with an in vitro model on CAL27 cell culture, we decided to construct an in vivo xenograft model of oral cancer to confirm the beneficial effect of BAY-117082. 

Our data demonstrate that BAY-117082 treatment significantly reduced subcutaneous tumor mass and tumor necrosis compared to the control group in a dose-dependent manner. Additionally, treatment with BAY-117082 significantly decreased tumor burden and tumor weight compared to the control group in a dose-dependent manner, without changing the animals’ weight. 

Moreover, considering the key role of NLRP3 inflammasome in oral carcinogenesis [20], we also investigated the effect of BAY-117082 in an in vivo xenograft model. Our results confirm that BAY-117082 was able to significantly reduce NLRP3 activation, also decreasing ASC and caspase-1 expression compared to the control group. Additionally, we investigated the expression of pro-inflammatory cytokine IL-1β, which is involved in the NLRP3 inflammasome pathway [39], showing that treatment with BAY-117082 was able to significantly reduce its expression in a dose-dependent manner. 

It has been demonstrated that the NF-κB signaling pathway has a fundamental role in oral cancer pathogenesis [24]. NF-κB is a transcription factor that regulates different biological processes, including cell growth, proliferation, and apoptosis [25]. However, abnormal activation of NF-κB contributes to the development of various autoimmune, inflammatory, and malignant diseases such as cancer, promoting metastasis and invasiveness [25]. Thus, in this study we decided to evaluate the NF-κB/IκB-α signaling pathway, demonstrating that treatment with BAY-117082 significantly reduced NF-κB translocation into the nucleus and restored IκB-α cytosolic expression in a dose-dependent manner. 

In addition, NF-κB plays a critical role in regulating the activation and differentiation of innate immune cells and inflammatory T cells [40]. In a malignant disease such as cancer, the response to chemotherapy is influenced by immune system activation, which can promote or attenuate tumorigenesis [26]. Recently, emphasis is increasingly being placed on the role of the immune system and its association with the occurrence and progression of cancer [41]. Despite the impressive successes in cancer immunotherapy, the response in patients is sometimes short-lived [41]. This is due to factors that hamper the immune response against cancer, such as the excessive and aberrant presence of CD4 T and CD8 T cells in the tumor microenvironment [41]. Therefore, based on these findings, we decided to investigate the effect of BAY-117082 on the immune system by evaluating CD4, CD8, and CD30 expression. In this context, our results demonstrate that BAY-117082 treatment reduced CD4, CD8, and CD30 levels compared to the control group in a dose-dependent manner. 

Furthermore, to confirm the data obtained in the in vitro model with regard to the apoptosis pathway, we decided to also investigate the effect of BAY-117082 on programmed cell death in the xenograft model, demonstrating that it was able to significantly increase pro-apoptotic Bax protein expression, while anti-apoptotic Bcl2 and Bcl-xL were significantly reduced following BAY-117082 treatment in a dose-dependent manner, in contrast to cell proliferation. 

Another important hallmark of cancer is cell-cycle dysregulation [42]. During oral carcinogenesis, growth signaling become dysregulated and cancer cells can proliferate without exogenous stimulation [43]. Ki-67 is a nuclear protein closely associated with cell proliferation [28]. It is exclusively present within the nucleus during the interphase, and since it is present during all phases of the cell cycle (G1, S, G2, mitosis), it may represent a useful marker of cell growth [44]. In the last decade, Ki-67 has been widely used as a prognostic marker for cancer progression [28]. 

Therefore, in this study we decided to investigate Ki-67 expression, showing that the control group was characterized by elevated Ki-67 expression, whereas treatment with BAY-117082 was able to significantly reduce its appearance. 

Thus, based on the results obtained, using BAY-117082 could be considered as a valid therapeutic strategy to reduce or counteract OSCC progression thanks its ability to modulate the NLRP3 inflammasome and apoptosis pathways. However, further investigations are needed to better understand the involvement of these pathways in oral carcinogenesis. 

## 4. Material and Methods

### 4.1. In Vitro Studies 

#### 4.1.1. Cell Culture

Human OSCC cell lines CAL27, HSC-2, and SCC-4 were obtained from ATCC (Manassas, VA, USA). Cells were grown in Dulbecco’s Modified Eagle’s Medium (Invitrogen, Waltham, MO, USA) for CAL27 and Minimum Essential Eagle’s Medium (Sigma-Aldrich, St. Louis, MO, USA) for HSC-2 and SCC-4 cells, supplemented with 10% fetal bovine serum (FBS) (Invitrogen) and 100 U/mL penicillin and 100 μg/mL streptomycin (Sigma-Aldrich, St. Louis, MO, USA) at 37 °C with 5% CO_2_.

#### 4.1.2. MTT Assay 

A 3-(4,5-dimethylthiazol-2-yl)-2,5-diphenyltetrazolium bromide (MTT) assay was used to assess cell viability, as previously described [45]. CAL27, HSC-2, and SCC-4 cells were plated on 96-well plates at a density of 4b × b10^4^ cells/well to a final volume of 150 μL. After 24 h, cells were treated with BAY-117082 (Sigma-Aldrich^®^) for 24 h at increasing concentrations 0.1, 0.5, 1, 3, 5, 10, and 30 μM dissolved in PBS. Then, cells were incubated at 37 °C with MTT (0.2 mg/mL) for 1 h. The medium was removed and cells were lysed with dimethyl sulfoxide (DMSO) (100 µL). The extent of reduction in MTT to formazan was quantified by measuring optical density at 550 nm with a microplate reader.

##### Experimental Groups

1. Control group (Ctr): Human OSCC cell lines CAL27, HSC-2, and SCC-4
2. BAY-117082 0.1 μM group: CAL27, HSC-2, and SCC-4 cells treated with BAY-117082 0.1 μM for 24 h
3. BAY-117082 0.5 μM group: CAL27, HSC-2, and SCC-4 cells treated with BAY-117082 0.5 μM for 24 h
4. BAY-117082 1 μM group: CAL27, HSC-2, and SCC-4 cells treated with BAY-117082 1 μM for 24 h
5. BAY-117082 3 μM group: CAL27, HSC-2, and SCC-4 cells treated with BAY-117082 3 μM for 24 h
6. BAY-117082 5 μM group: CAL27, HSC-2, and SCC-4 cells treated with BAY-117082 5 μM for 24 h
7. BAY-117082 10 μM group: CAL27, HSC-2, and SCC-4 cells treated with BAY-117082 10 μM for 24 h
8. BAY-117082 30 μM group: CAL27, HSC-2, and SCC-4 cells treated with BAY-117082 30 μM for 24 h

#### 4.1.3. Western Blot Analysis of NLRP3, ASC, Caspase-1, IL-1β, IL-18, Bax, Bcl2, and Bad 

For the Western blot, 1 × 10^6^ CAL27 cells were plated in 6-well plates (Corning Cell Culture, Tewksuby, MA, USA) and incubated with BAY-117082 for 24 h [45]. Then, cells were washed with phosphate buffered saline (PBS), scraped, and pelleted for protein lysate preparation. The CAL27 cells were resuspended in 20 mM Tris-HCl, pH 7.5, 10 mM NaF, 150 mM NaCl, 1% Nonidet P-40, and protease inhibitor cocktail (Roche, Basel, Switzerland). After 40 min, cell lysates were centrifuged at 16,000× *g* for 15 min at 4 °C. Protein concentration was estimated by the Bio-Rad protein assay using bovine serum albumin as standard. Samples were heated at 95 °C for 5 min, and the same amounts of protein were separated on 12% SDS-PAGE gel and blotted to PVDF membrane (Immobilon-P). Membranes were incubated overnight at 4 °C with the following primary antibodies: anti-NLRP3 (sc-34411, 1:500; Santa Cruz Biotechnology, Dallas, TX, USA), anti-ASC (sc-22514, 1:500; Santa Cruz Biotechnology, Dallas, TX, USA), anti-caspase-1 (sc-514, 1:500; Santa Cruz Biotechnology, Dallas, TX, USA), anti-IL-1β (sc-32294, 1:500; Santa Cruz Biotechnology, Dallas, TX, USA), anti-IL-18 (sc-80051, 1:500; Santa Cruz Biotechnology, Dallas, TX, USA), anti-Bax (sc-7480, 1:500; Santa Cruz Biotechnology, Dallas, TX, USA), anti-Bcl2 (sc-7382, 1:500; Santa Cruz Biotechnology, Dallas, TX, USA), and anti-Bad (sc-8044, 1:500; Santa Cruz Biotechnology, Dallas, TX, USA). Then, membranes were incubated with peroxidase-conjugated bovine anti-mouse or goat anti-rabbit or anti-mouse IgG (1:2000; Jackson ImmunoResearch, Jackson Laboratories, Bar Harbor, ME, USA) for 1 h at room temperature. To ascertain that the blots were loaded with equal amounts of proteins, they were also incubated in the presence of the antibody against β-actin protein for cytosolic fraction (1:500; Santa Cruz Biotechnology) and Lamin A/C for nuclear fraction (1:500; Santa Cruz Biotechnology). The signals were detected with a chemiluminescence detection system reagent according to the manufacturer’s instructions (Super Signal West Pico Chemiluminescent Substrate, Pierce Thermo Scientific, Rockford, IL, USA). The relative expression of protein bands was quantified by densitometry with Bio-Rad ChemiDoc (Bio-Rad, Genzano di Roma, Rome, Italy) using ImageLab software.

#### 4.1.4. Immunofluorescence Assay for p53 and Caspase-1

Immunofluorescence assay was performed as previously described by Donaldson [46]. CAL27 cells on glass coverslips were rinsed briefly in PBS (0.15 M NaCl, 10 mM Na_2_HPO_4_, pH 7.4), permeabilized in 0.2% Triton X-100/PBS, and blocked with 10% bovine albumin serum. Cells were incubated overnight (O/N) at 4 °C with primary antibodies: anti-p53 (sc-126, 1:100; Santa Cruz Biotechnology, Dallas, TX, USA) and anti-caspase-1 (sc-514, 1:100; Santa Cruz Biotechnology, Dallas, TX, USA). After being washed in PBS, cells were incubated with secondary antibody Alexa Fluor 488 goat anti-mouse (1:1000 *v/v*; Molecular Probes, cat # A32723, MCR, UK) for 1 h at 37 °C. Sections were washed in PBS and 2 μg/mL 4′,6′-diamidino-2-phenylindole (DAPI; Hoechst, Frankfurt, Germany) in PBS was added for nuclear staining. Sections were observed and photographed at 40× magnification using a Leica DM2000 microscope (Leica, Axiostar plus). 

### 4.2. In Vivo Studies

#### 4.2.1. Animals

BALB/c nude male mice were obtained from Jackson Laboratory (Bar Harbor, ME, USA) and housed in microisolator cages under pathogen-free conditions with 12 h light/12 h dark. Animals were fed with a standard diet and water ad libitum. This study was approved by the University of Messina Review Board, under project identification code 137/2017-PR, released on 9 February 2017. Animal care was in compliance with Italian regulations on the protection of animals used for experimental and other scientific purposes (DM 116192) as well as EU regulations (OJ of EC L 358/1 18 December 1986).

#### 4.2.2. Xenograft Tumor Model

The xenograft tumor model was established by subcutaneously inoculating 3 × 10^6^ CAL27 cells per tumor in 0.2 mL of PBS and 0.1 mL Matrigel (BD Bioscience, Bedford, MA, USA) as previously described [47]. After tumor cell inoculation, animals were monitored daily for morbidity and mortality, and their body weight was monitored weekly to evaluate overall health. 

After 1 week of tumor induction, mice were divided randomly into 3 groups. When tumor size reached about 200–300 mm^3^, mice were treated with BAY-117082 at doses of 2.5 and 5 mg/kg every 3 days according to [17,48]. BAY-117082 was dissolved in PBS with 0.001% DMSO. The tumor size was monitored daily by a caliper and calculated as follows: V = W^2^ × L/2, where W and L represent minor and major length. After 30 days, mice were sacrificed and tumors were excised and processed for analysis. 

##### Experimental Groups

Mice were randomly divided into 3 groups:

1. Control group (vehicle): weekly intravenous (IV) administration of saline
2. Control group + BAY-117082 2.5 mg/kg: intraperitoneal administration of BAY-117082 2.5 mg/kg dissolved in PBS
3. Control group + BAY-117082 5 mg/kg: intraperitoneal administration of BAY-117082 5 mg/kg dissolved in PBS

The minimum number of mice for each technique was estimated with one-way fixed effects ANOVA with G-power software. This statistical test generated a sample size equal to *n* = 14 mice for each technique. 

#### 4.2.3. Histological Evaluation

Histological evaluation was performed as previously described by Paterniti et al. [49]. Tumor samples were quickly removed and fixed with 10% buffered formalin for at least 24 h at room temperature. After dehydration in graded ethanol and xylene, tumor samples were embedded in paraffin and sectioned at 7 μm thickness. After staining with hematoxylin and eosin, sections were observed by an optical microscope (Axostar Plus equipped with Axio-Cam MRc, Zeiss, GE, Germany). The histological results are shown at 20× and 40× magnification (bar scale at 50 and 20 μm, respectively). All histological analyses were executed in a blinded manner.

#### 4.2.4. Immunohistochemical Localization of IL-1β, Ki-67, CD4, CD8, and CD30

Immunohistochemical localization was carried out as previously described by Scuderi et al. [6]. Tumor sections were incubated overnight at room temperature with the following primary antibodies: anti-Ki-67 (sc-23900, 1:100; Santa Cruz Biotechnology, Dallas, TX, USA), anti-IL-1β (sc-32294, 1:100; Santa Cruz Biotechnology, Dallas, TX, USA), anti-CD4 (sc-13573, 1:100; Santa Cruz Biotechnology, Dallas, TX, USA), anti-CD8 (sc-1177, 1:100; Santa Cruz Biotechnology, Dallas, TX, USA), and anti-CD30 (sc-19984, 1:100; Santa Cruz Biotechnology, Dallas, TX, USA). After this incubation, the sections were washed with PBS and incubated with a secondary antibody (Santa Cruz Biotechnology, Santa Cruz, CA, USA) for 1 h. The reaction was revealed by a chromogenic substrate (brown DAB), and counterstaining with Nuclear Fast Red. A negative control was performed using no primary antibody; specifically, tissue was incubated with the antibody diluent alone, followed by incubation with secondary antibodies and detection reagents. For immunohistochemistry, 20× (50 µm scale bar) and 40× (20 µm scale bar) magnification are shown.

#### 4.2.5. Western Blot Analysis of NLRP3, ASC, Caspase-1, NF-κB, IκB-α, Bax, Bcl2, and Bcl-xL

Protein levels in tumor samples were quantified as previously described [6]. Cytosolic proteins were prepared and separated electrophoretically to be transferred to nitrocellulose membranes. Membranes were blocked with 5% (*w/v*) dried nonfat milk in buffered saline (PM) for 45 min at room temperature and subsequently probed with specific antibodies: anti-NLRP3 (sc-34411, 1:500 Santa Cruz Biotechnology, Dallas, TX, USA), anti-ASC (sc-22514, 1:500; Santa Cruz Biotechnology, Dallas, TX, USA), anti-caspase-1 (sc-514, 1:500; Santa Cruz Biotechnology, Dallas, TX, USA), anti-Bax (sc-7480, 1:500; Santa Cruz Biotechnology, Dallas, TX, USA), anti-Bcl2 (sc-7382, 1:500; Santa Cruz Biotechnology, Dallas, TX, USA), anti-Bcl-xL (sc-8392, 1:500; Santa Cruz Biotechnology, Dallas, TX, USA), anti-NF-κB (sc-8008, 1:500, Santa Cruz Biotechnology, Dallas, TX, USA), and anti-IκBα (sc-1643, 1:500; Santa Cruz Biotechnology, Dallas, TX, USA) in 1× PBS, 5% *w/v* dried nonfat milk and 0.1% Tween-20 (PMT) at 4 °C overnight. Membranes were incubated with peroxidase-conjugated goat anti-mouse IgG secondary antibody (1:2000, Jackson ImmunoResearch, West Grove, PA, USA) or peroxidase-conjugated goat anti-rabbit IgG secondary antibody (1:5000, Jackson ImmunoResearch, West Grove, PA, USA) for 1 h at room temperature. To establish that blots were loaded with equal amounts of proteins, they were also incubated in the presence of the antibody against β-actin protein (sc-8432, 1:500; Santa Cruz Biotechnology, Dallas, TX, USA). Signals were revealed with an enhanced chemiluminescence (ECL) detection system reagent according to the manufacturer’s instructions (Thermo, Waltham, MO, USA, cat# 457). The relative expression of protein bands was quantified by densitometry with Bio-Rad ChemiDoc XRS+ software and standardized to β-actin levels as an internal control.

### 4.3. Materials

All compounds and other chemicals were obtained from Sigma-Aldrich (Milan, Italy). All stock solutions were prepared in non-pyrogenic saline (0.9% NaCl; Baxter, Milan, Italy).

### 4.4. Statistical Analysis

Data were analyzed with GraphPad Prism 7.04 software using *t*-test analysis. All values are indicated as mean ± standard error of the mean (SEM) of N observations. 

## 5. Conclusions

In conclusion, the results obtained offer new insights into the role of the NLRP3 inflammasome and apoptosis signaling pathways in oral carcinogenesis, showing that the use of BAY-117082, a strong NLRP3 inflammasome inhibitor, could represent a potential therapeutic treatment to counteract or reduce the progression of OSCC, which has been showing an increasing mortality rate. 

## Data Availability

The authors declare that all data and materials supporting the findings of this study are available within the article. The data that support the findings of this study are available from the corresponding author upon reasonable request.
